# Characteristics of Medical Research News Reported on Front Pages of Newspapers

**DOI:** 10.1371/journal.pone.0006103

**Published:** 2009-07-01

**Authors:** William Yuk Yeu Lai, Trevor Lane

**Affiliations:** 1 Journalism and Media Studies Centre, The University of Hong Kong, Pokfulam, Hong Kong, People's Republic of China; 2 Faculty of Dentistry, The University of Hong Kong, Prince Philip Dental Hospital, Hong Kong, People's Republic of China; Science Commons, United States of America

## Abstract

**Background:**

The placement of medical research news on a newspaper's front page is intended to gain the public's attention, so it is important to understand the source of the news in terms of research maturity and evidence level.

**Methodology/Principal Findings:**

We searched LexisNexis to identify medical research reported on front pages of major newspapers published from January 1, 2000 to December 31, 2002. We used MEDLINE and *Google Scholar* to find journal articles corresponding to the research, and determined their evidence level.

Of 734 front-page medical research stories identified, 417 (57%) referred to mature research published in peer-reviewed journals. The remaining 317 stories referred to preliminary findings presented at scientific or press meetings; 144 (45%) of those stories mentioned studies that later matured (i.e. were published in journals within 3 years after news coverage). The evidence-level distribution of the 515 journal articles quoted in news stories reporting on mature research (3% level I, 21% level II, 42% level III, 4% level IV, and 31% level V) differed from that of the 170 reports of preliminary research that later matured (1%, 19%, 35%, 12%, and 33%, respectively; chi-square test, *P* = .0009). No news stories indicated evidence level. Fewer than 1 in 5 news stories reporting preliminary findings acknowledged the preliminary nature of their content.

**Conclusions/Significance:**

Only 57% of front-page stories reporting on medical research are based on mature research, which tends to have a higher evidence level than research with preliminary findings. Medical research news should be clearly referenced and state the evidence level and limitations to inform the public of the maturity and quality of the source.

## Introduction

Popular media such as newspapers are commonly the initial source of medical research news for both medical professionals and the public [1]–[4]. Medical research news on the front pages of newspapers in particular needs to be reliable because of the intended maximum or immediate impact on the reader. If not carefully prepared, front-page news about medical research can have powerful, undesirable consequences such as scaremongering and misinformation [5].

To medical researchers and health care professionals, the most credible source of medical information is generally accepted to be “mature” research, that is, studies published in peer-reviewed journals [6]. Among the reasons are the following: Firstly, although peer review is imperfect [7], [8]—most notably, it cannot prevent research fraud [9]—it is the standard process by which independent experts vet the reliability and validity of submitted research, by scrutinizing aspects such as methodology, analysis, and interpretation. Secondly, publication status indicates the “maturity” of research, and journal publication often represents the formal end-point of a research study. Medical research news based on published studies can thus be regarded as being more credible than news generated from other sources, such as scientific or press meetings presenting preliminary findings that are not yet published [10], [11]. Finally, journal publication creates a retrievable archive of medical information that can be evaluated for quality and usefulness, as reflected by the strength of the presented evidence (e.g. study design, sample size, and clinical relevance). Appraisal of this aspect of medical studies—the basis of evidence-based medicine (EBM)—helps health care practitioners to identify, filter, and apply the current best evidence to solve specific clinical problems [12], [13].

The maturity and evidence level of medical research reported on newspaper front pages have so far not yet been investigated. We therefore aimed to systematically characterize sources of front-page medical research news in terms of journal publication status and level of evidence, and to assess whether these characteristics were reported in news stories.

## Methods

### Data Sample and Study Maturity

We searched LexisNexis, an electronic news archive, for medical and health stories published on the front pages of any section of newspapers during a 3-year period from January 1, 2000 to December 31, 2002. In the “Major Papers” domain, which covers more than 50 high-circulation, English-language national and regional newspapers worldwide, we used “pg. 1”, “pg. A1”, “pg. 1A”, or “pg. A01” in the “full-text” field and the keyword “medical” or “health” in the default “headline, lead paragraph(s), terms” field. To target front-page news about medical research studies (e.g. treatments, drugs, medical devices, and public health developments), we refined our search by using the additional keyword “study” or “studies.” Next, the retrieved news items were manually searched to identify the source of medical research news and to exclude stories that were not truly based on studies with research findings (e.g. business and financial reports, medico-legal analyses, medical policies, health insurance stories, animal activist protests, and announcements of upcoming conferences and future studies) and duplicate news stories originating from different daily editions of the same newspaper.

Front-page news stories were classified according to whether or not they mentioned research already published in peer-reviewed journals. We assumed that peer review is the globally accepted quality-control process for biomedical journals and that studies published in journals are mature, reliable sources of medical information. In contrast, studies with preliminary findings were defined as those quoted in newspapers that had not yet been published in a journal but had been presented in scientific or press meetings, such as conferences convened by professional or academic organizations, or those initiated by the general press, private institutions, commercial organizations and public relations companies. Also included in this category were “tip offs” and anecdotal evidence mentioned by individuals within the medical community. We excluded news stories that were based on surveys or reports that had been compiled by government agencies, such as local, regional, state, or national governments and government departments (e.g. US Environmental Protection Agency), by international government-related organizations (e.g. United Nations Children's Fund), and by nongovernment agency and charity groups, because the publication status, peer review status, and maturity were usually unclear or impossible to trace.

For news items quoting mature research but not directly citing a journal, we checked whether the source had been published in a peer-reviewed journal by searching for stated names of researchers or topic keywords in MEDLINE and *Google Scholar*. Furthermore, we expected that some preliminary findings would mature, that is, they would be published later in peer-reviewed journals [11]. We thus subcategorized quoted studies presenting preliminary findings by whether they were subsequently published in journals, as deduced from MEDLINE and *Google Scholar* searches. We used a 3-year follow-up period because we expected 95% of publishable studies originating from scientific conferences to be published within this period [11], [14], [15].

**Figure 1:**
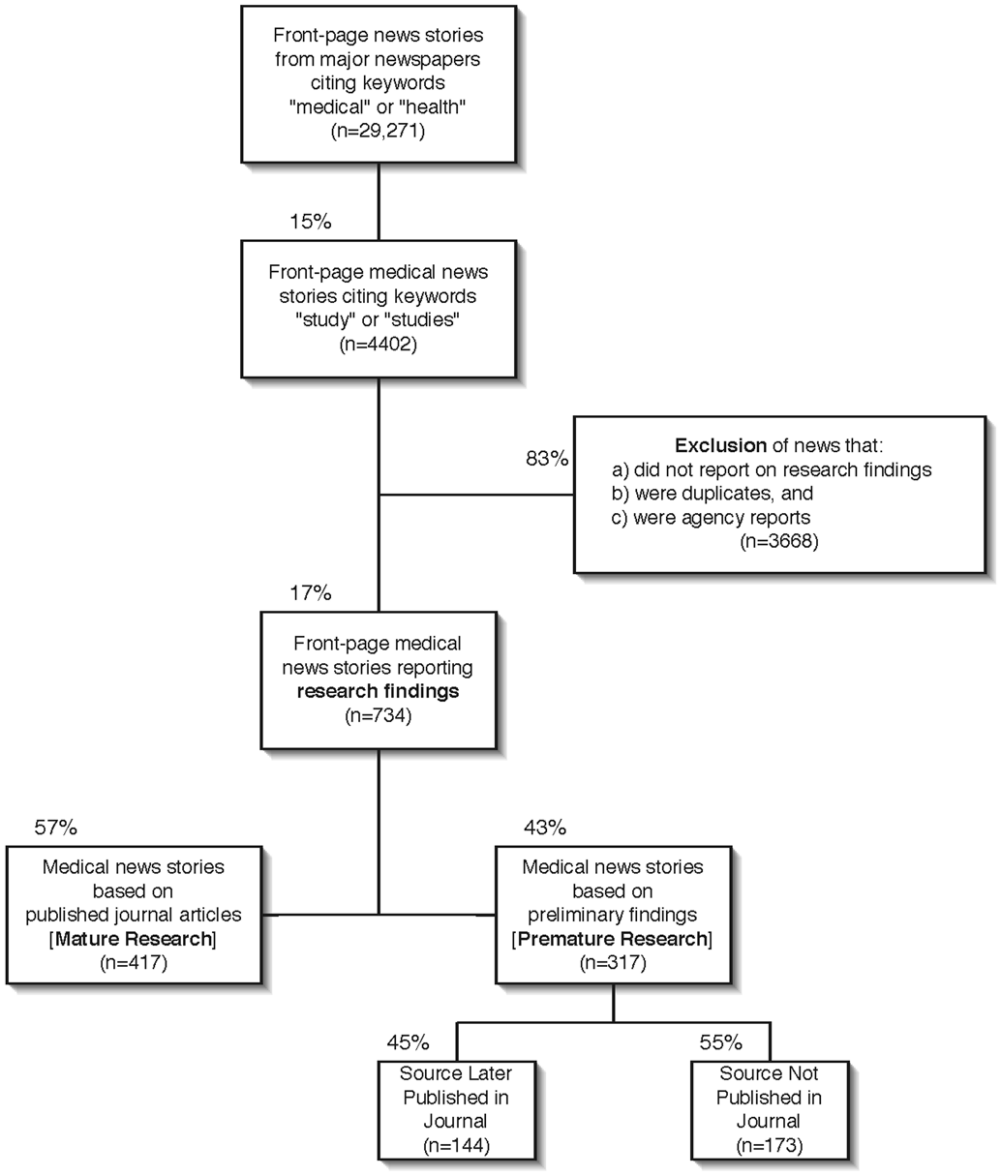
Categorization of front-page medical news stories between 2000 and 2002 that reported research findings.

The **Figure 1** shows how newspaper front-page stories on medical research were selected and categorized. Any newspapers based on “early release” journal articles (i.e. early online publication or Epub) were classified as quoting mature research. Occasionally a front-page story referenced a study that had been both presented at a meeting and already published in a peer-reviewed journal; the news source was then classified as mature. When a story quoted both mature and preliminary research findings, the overall news source was classified as mature. For news stories reporting on more than one study with preliminary findings but at least one of which was published in a journal within 3 years after news coverage, the overall news source was classified as preliminary research that later matured.

### Evidence Levels

Next, we used abstracts of retrieved journal articles corresponding to the research reported in the news story to categorize the study design and hence the study's likely level of evidence. If needed, full papers were used to confirm the study design and estimate the evidence level. We modified the Oxford EBM classification system [13] and the Australian National Health and Medical Research guidelines [16] to accommodate 5 levels of evidence. Level I evidence was that contained in systematic reviews (SRs) of randomized controlled trials (RCTs). Level II evidence was contained in RCTs (we included all RCTs rather than just good-quality ones). Level III evidence comprised prospective and retrospective cohort studies, ecological studies, and case-control studies, as well as SRs not of RCTs. Level IV evidence came from case series, and level V evidence came from expert opinions (case reports, descriptive or methodological studies, narrative or nonsystematic reviews, qualitative studies, audits, reports of expert committees, research letters, technical notes, and tutorials) and animal or laboratory studies. We excluded commentaries, editorials, news items, and nonresearch letters from EBM classification because such articles do not usually contain research evidence, are not always peer-reviewed, and the first 3 types are usually commissioned by journal editors.

### Research Topic

We classified the topic of each journal article using an expanded range of focus topics used by Bartlett et al [17]. When classification was not possible from the information provided in an abstract, we examined the full journal article.

### Statistical Analysis

All source classifications according to publication status, level of evidence, study type, and research topic were done by one researcher. A second researcher coded a 20% random sample of news stories. Intercoder reliability (kappa) was calculated with SPSS version 13.0 (SPSS Inc, Chicago, Illinois). A score of 0.7 and higher indicates an acceptable degree of intercoder reliability. Chi-square tests were performed on categorical data with JMP version 5.1 (SAS Institute, Cary, NC). A *P* value of ≤.05 was considered statistically significant.

## Results

Of 734 front-page medical research news stories that mentioned study findings, 417 (57%) were based on mature research and 317 (43%) were based on preliminary findings at the time of news coverage (**Figure 1**). The 734 front-page medical research stories came from 47 different English-language newspapers over a 3-year period (**Table S1**). Kappa scores for maturity status of reported research and source of research information used were 0.847 and 0.926, respectively.

### Front-page Medical Research News Based on Mature Studies

The 417 front-page stories reporting on mature research were each based on findings from 1 to 4 published studies, with a total of 567 mentions of research studies published in 435 distinct journal articles. Of the 417 news stories, 251 did not overlap in content and mentioned 333 journal articles once: 190 stories reported on one journal article, 45 reported on 2 different articles, 11 reported on 3 articles, and 5 reported on 4 articles. Among the 166 news stories that showed some overlap in content, 113 reported on one journal article, 38 reported on 2 articles, and 15 reported on 3 articles. Twenty-seven journal articles were quoted once and 75 articles were quoted from 2 to 31 times (total, 234 article mentions). For example, an article on the Women's Health Initiative trial [18] was quoted in 31 front-page news stories.

The majority of the 417 news stories simply reported study findings, but 10 (2%) stories also challenged the findings of the published research. Only 101 (24%) news stories correctly stated the study type, mostly animal or laboratory studies; the rest did not report study type. None explicitly reported evidence level. Among the 567 newspaper mentions of any findings published in journals, 464 (82%) referenced the journal source correctly, 8 (1%) were referenced incorrectly, and 95 (17%) were not referenced.

For the analyses of evidence levels and study types, we used as the denominator all mentions of published findings (i.e. weighted citations) after excluding articles not presenting any evidence (50 commentaries or editorials and 2 nonresearch letters). The 515 mentions of mature findings were most commonly associated with level III and V evidence (42% and 31%, respectively) and least commonly with level I evidence (3%) [**Table 1**]. Observational study (OS) types were the most commonly reported, together accounting for about half of the mature research cited (46%, level III and IV evidence). Animal and laboratory studies made up 56% (88/158) of mature research presenting level V evidence and 17% (88/515) of all quoted mature research. The 515 mature research studies came from a total of 122 different journals (**Table S2**). Quoted articles most commonly came from the *Journal of the American Medical Association* (104/515; 20%) [**Table 2**].

**Table 1:** Evidence Level and Research Topic of Medical Research Articles Published Before or After News Coverage*.

	BEFORE, No. (%) (n = 515)	AFTER, No. (%) (n = 170)	*P* value
**Evidence level** † **and study design:**			
I: Systematic reviews of randomized controlled trials	14 (3)	1 (1)	(Chi-square test) .0009
II: Randomized controlled trials	106 (21)	33 (19)	
III: Prospective/retrospective cohort studies, ecological studies, case-control studies, and systematic reviews not of randomized controlled trials	217 (42)	59 (35)	
IV: Case series	20 (4)	21 (12)	
V: Expert opinions‡ and animal/laboratory studies	158 (31)	56 (33)	
**Research Topic:**			
Women's health	126 (24)	17 (10)	na
Paediatrics	57 (11)	17 (10)	
Men's health	4 (1)	8 (5)	
Old age	9 (2)	1 (1)	
Cancer	20 (4)	21 (12)	
Heart disease	48 (9)	20 (12)	
Diabetes	10 (2)	1 (1)	
Reproduction	16 (3)	1 (1)	
Mental health	33 (6)	14 (8)	
Public, environmental & occupational health	96 (19)	29 (17)	
Infectious diseases	33 (6)	16 (9)	
Other medical research§	63 (12)	25 (15)	

* Denominators are weighted and are any article used as a front-page news source (Before) and any article published within 3 years of news coverage corresponding to any preliminary study used as a front-page news source (After).

† Modified from the Oxford EBM classification system [13] and the National Health and Medical Research guidelines [16].

‡ Expert opinions comprised case reports, descriptive/methodological studies, narrative reviews, qualitative studies, audits, reports of expert committees, research letters, technical notes, and tutorials. Commentaries or editorials, news items, and nonresearch letters from journals were excluded from analysis (52 from “Before” and 7 from “After”).

§ Studies not classifiable using the other 11 topics (e.g. bionic eye studies, bone cement research).

na = not applicable.

**Table 2:** Biomedical Journals Most Commonly Publishing Medical Research Articles Related to Front-page Medical News Stories.

Journal	No. (%)
**Published Before News Coverage** * **(n = 515)**	
*1. Journal of the American Medical Association*	104 (20)
*2. New England Journal of Medicine*	71 (14)
*3. Lancet*	36 (7)
*4. Nature*	28 (5)
*5. Science*	25 (5)
*6. BMJ*	20 (4)
*7. New Zealand Medical Journal*	17 (3)
*8. Journal of the National Cancer Institute*	10 (2)
*9. Pediatrics*	10 (2)
*10. Medical Journal of Australia*	7 (1)
**Published After News Coverage** † **(n = 170)**	
*1. Lancet*	9 (5)
*2. New England Journal of Medicine*	8 (5)
*3. Journal of Clinical Oncology*	5 (3)
*4. Nature*	5 (3)
*5. Science*	5 (3)

* Denominator is any article used as a front-page news source (weighted).

† Denominator is any article published within 3 years of news coverage corresponding to any preliminary findings used as a front-page news source (weighted). Only the 5 most common journals are shown.

### Front-page Medical Research News Based on Preliminary Findings

A total of 317 front-page stories were each based on preliminary findings from 1 to 5 studies, with a total of 365 mentions of research from 337 distinct studies. There were 274 stories with distinct sources (total, 316 studies) and 43 overlapping stories that were based on 21 studies (3 studies were reported 4 times, one was reported 3 times, and 17 were reported twice; total, 49 mentions). Only 116 (37%) news stories stated the name of the meeting or organizers and only 56 (18%) specifically stated that the findings were preliminary, had been released early, or had limitations because they came from unpublished research. None reported the evidence level.

For more than half (55%; 173/317) of the news stories that were based on preliminary findings, or 24% (173/734) of all stories, the corresponding studies (188 in total) remained unpublished within 3 years. In each of the remaining news stories reporting on preliminary findings (45%; 144/317), at least one of the quoted studies matured within 3 years after news coverage by publication in a peer-reviewed journal. Of these 144 news stories, 114 reported on one study, 21 reported on 2 studies, 6 reported on 3 studies, 2 reported on 4 studies, and one reported on 5 studies. The total number of newspaper mentions of studies with preliminary findings that later matured was 177, but the actual number of distinct studies was 157.

For the analyses of evidence levels and study types of research that matured after newspaper coverage, we again used as the denominator published articles corresponding to all mentions of studies (i.e. weighted citations) after excluding articles not presenting evidence (5 commentaries or editorials, 1 nonresearch letter, and 1 news item). The 170 articles again most commonly contained level III or V evidence (35% and 33%, respectively) and least commonly level I evidence (1%) [**Table 1**]. The most frequently used design was again the OS, accounting for about half of the articles (47%, level III and IV evidence). Animal and laboratory studies made up 61% (34/56) of papers presenting level V evidence and 20% (34/170) of the total. The mean interval between news coverage and publication of a study in a journal was 15.9 months (95% CI: 14.4 to 17.5 months) and the median interval was 14.0 months (range, 1.0 to 36.0 months). The articles appeared in 113 different journals (**Table S3**), most commonly the *Lancet* (9/170; 5%) [**Table 2**].

### Comparison of Studies Published Before and After News Coverage

When we examined the study designs of the 515 mentioned studies that had already been published at the time of news coverage, we found that 46% were OSs, 21% were RCTs, 17% were animal or laboratory studies, 14% were expert opinions, and 3% were SRs of RCTs. This profile was similar to that of the 170 cited studies with preliminary findings that later matured (47%, 19%, 20%, 13%, and 1%, respectively; chi-square test, *P* = .3826). However, when the EBM levels were examined, the evidence-level distribution of the 515 mature research papers quoted in news stories (3% level I, 21% level II, 42% level III, 4% level IV, and 31% level V) was different from that of the 170 studies with preliminary findings that later matured (1%, 19%, 35%, 12%, and 33%, respectively; chi-square test, *P* = .0009). [**Table 1**].

### Research Topic

Among the 515 mentioned studies that had already been published at the time of news coverage, the 3 leading research topics were women's health (126; 24%); public, environmental and occupational health (96; 19%); and “other” medical research (63; 12%). The 3 most common topics among the 170 cited studies with preliminary findings that later matured were public, environmental and occupational health (29; 17%); “other” medical research (25; 15%); and cancer (21; 12%) [**Table 1**].

## Discussion

This is the first study to examine the characteristics of a cross-section of front-page medical research news in major newspapers in terms of research maturity and associated level of scientific evidence. Only 57% of front-page news stories reporting medical research can be said to be based on mature research, whereas the remaining 43% cites research based on unpublished medical information, which is less easy to verify, reference, and assess. We recommend that journalists recognize and clearly distinguish between these 2 main kinds of medical information, because studies published in peer-reviewed medical journals have supposedly been scrutinized by experts, whereas unpublished studies are deemed by the scientific community to be preliminary and are not guaranteed to have undergone a rigorous and independent review process. Peer review would also ideally ensure adherence to publishing guidelines such as the “Uniform Requirements” of the International Committee of Medical Journal Editors [19] and reporting guidelines promoted by the EQUATOR Network [20], and journals may attempt to further improve papers before publication through technical editing [21]. Journalists should therefore try to avoid rushing to report preliminary findings [11], [22], [23], as this would allow time for confirmation of results, preparation for journal submission, and scrutiny and revision during peer review. Furthermore, being patient before reporting medical research until it has been published in a peer-reviewed journal should help increase public confidence in the media and reduce occurrences of misinformation.

We acknowledge that the journalistic process may emphasize being first to report a news story, making conferences a popular information source. Still, Woloshin and Schwartz [23] found that news stories reporting preliminary research findings often missed basic study facts and rarely noted the preliminary nature of the research presented. In our analysis, fewer than 1 in 5 news stories reporting preliminary findings acknowledged the preliminary nature of their content. Our study also revealed that 55% of news stories quoting preliminary findings (24% of all stories) mentioned studies that remained unpublished in the 3 years after reporting. It could be argued that the quality of such research is inferior to that of preliminary research that is subsequently published. In a study tracking the fate of research reported in abstracts at particular scientific meetings, Schwartz et al [11] found that one-quarter of abstracts that received media coverage remained unpublished 3.5 years after the meeting. Those authors also suggested a difference in research quality, but acknowledged that subsequent publication may be an imperfect indicator of scientific quality, because some research may never be submitted for publication and the quality of an accepted article for publication depends on the stringency of a journal's peer review process. Nevertheless, if reporters do choose to base front-page news stories on research that is preliminary, we recommend that they always state any study limitations and point out that preliminary findings and interpretations require verification and confirmation.

We did not detect a difference between the distributions of study types among research that was mature at the time of news coverage and research that matured later. Assuming research articles published after news coverage retain their original study design, the most frequently reported study types on newspaper front pages overall were OSs, followed by RCTs, animal or laboratory studies, expert opinions, and SRs. Bartlett et al [17] also found that newspapers most commonly used OSs (58% OSs, 31% “others,” 6% RCTs, and 5% SRs among 81 newspaper medical stories). Unlike in our study, they found that articles in the “others” category (equivalent to our “expert opinions” category) were reported quite frequently, perhaps because all page positions were included in their analysis. Our data, however, show that expert opinions do not receive prominent coverage on front pages, which is both understandable and desirable.

The predominance of OSs among studies reported in front-page news stories may indicate either that the media prefer to report this type of study or that OSs are overrepresented among the information sources available to journalists, both in journals and at meetings, whereas SRs and RCTs are underrepresented. Bartlett et al [17] found that RCTs from *BMJ* and the *Lancet* were significantly underreported in newspapers, when they compared the proportions of published RCTs and OSs that were selected for news coverage (2%; 5/295 vs 11%; 47/444). Yet, the authors noted that those 2 medical journals issued press releases of RCTs and OSs in similar proportions (45%; 133/295 and 49%; 219/444, respectively) [17], suggesting that definitions of newsworthiness among journal public relations departments and journal editors differ from those among newspaper reporters and editors.

Our analysis comparing research that matured before and after newspaper coverage suggests that mature medical research chosen to appear on front pages generally has a higher evidence level than preliminary research. Assuming research articles published after news coverage retain their original evidence level, we found that level IV evidence tends to appear more frequently in front-page news based on preliminary findings than in news based on mature research; level III evidence tends to appear more frequently in the latter. Because both levels III and IV correspond to OSs, including EBM classification as well as study design type in news stories could provide extra, useful information about their sources. In our sample of newspaper front pages, only one-quarter of stories based on mature research evidence stated the study design correctly; none of the stories, whether based on mature or preliminary studies, stated evidence level or referred to any EBM classification. Hence, we recommend that reporters state and explain the level of evidence of any source study, although it is also important to realize that the best available evidence may not necessarily always be from SRs and RCTs (i.e. levels I & II, respectively), for logistic or ethical reasons. Medical professionals could assist the media by helping to put their research studies into context, clarifying risks [24], [25], and clearly explaining the current best medical evidence. Some medical journals (e.g. *Obstetrics and Gynecology*, *Journal of Bone and Joint Surgery*) encourage authors to indicate the level of evidence in their abstracts. If this becomes accepted practice among journals, then the media could also reflect this in the way they report medical research news.

Nearly 1 in 5 news stories based on mature research did not give a journal reference and only 2 in 5 news stories based on preliminary findings stated the information source. Clear citation of sources is essential if the media wish to convey accuracy and trustworthiness of medical information. This practice also allows readers to verify the quality of medical research news, especially because news stories may sometimes challenge the findings of published journal papers, as we noted in a small proportion of stories. Healthy debate of peer-reviewed research is to be encouraged, as long as such news stories help readers identify the current best evidence and clarify the context of findings without prioritizing selected opinions over others or over conclusions of published studies [26].

Finally, medical research news dealing with public, environmental and occupational health was often covered on front pages by the press. This trend may be related to the underlying preference for topics of research covered in OSs. Alternatively, perhaps the public tends to be interested in issues of public, environmental and occupational health and the majority of these studies are observational. Also, the widespread news coverage of the Women's Health Initiative trial [18] accentuated our study's profile of women's health and demonstrates the influence that a single journal article can have on news coverage [27]. Further studies may reveal whether the media have preferences in reporting certain health topics and if the preferences are related to the maturity and quality of the data source.

### Strengths and limitations of the study

An important strength of our study is the systematic selection of a large number of front-page medical research stories reporting on research findings in 47 English-language newspapers over a 3-year period. However, we noted the absence of some high-profile newspapers—*Baltimore Sun*, *Los Angeles Times*, and *The Times* (London)—on the LexisNexis source list; the first two were unavailable on LexisNexis within the study period of 2000–2002, and the third's front-page stories could not be distinguished from other pages. There is also a bias toward US newspapers. Our selection of front-page medical stories may therefore be representative of only major newspapers that agreed to archive stories in LexisNexis, were not date-restricted, and did not have variable page numbering. Furthermore, the final search of retrieved stories was based on the keywords “study” and “studies” for practical reasons; a future exhaustive study could use an array of synonyms, as well as confirm from principal investigators of preliminary research whether their study had been published in a peer-reviewed journal not appearing in MEDLINE or *Google Scholar*. Finally, our classifications of published studies relied mainly on checking journal abstracts, but the accuracy of data presented in abstracts may be deficient and even inconsistent with corresponding data presented in the full journal article [28].

### Conclusions

In our sample, only 57% of medical research news stories appearing on newspaper front pages were based on mature research and hence had been scrutinized by peer reviewers and made accessible to others for verification; 24% of news stories were based on preliminary findings that remained unpublished in the following 3 years. We encourage journalists to clearly distinguish whether medical research news is based on mature research or studies with preliminary findings; reference and cite sources well; and become familiar with EBM principles, especially when preparing front-page and hence high-profile medical news stories. Medical reporters and media organizations could enhance their reputations by advocating responsible medical news reporting, including mentioning evidence levels and study limitations [29]. Ultimately, researchers and reporters must work together on improving communication of medical research news, and researchers who present preliminary research at scientific and press conferences should routinely and explicitly include limitations and an appraisal of the level of evidence presented. Recently, a growing number of initiatives such as the *Hitting the Headlines* and *Media Doctor* websites are diligently monitoring the quality of medical news stories [30]–[33]. We hope such initiatives will inform the medical news reporting procedure.

## Supporting Information

Table S1 List of newspapers used in this study
(0.06 MB DOC)

Table S2 List of journals associated with news based on mature research
(0.11 MB DOC)

Table S3 List of journals associated with news based on preliminary findings
(0.10 MB DOC)
