# Supplementary material for: Characteristics of Medical Research News Reported on Front Pages of Newspapers
Source: PLoS One. 2009 Jul 1;4(7):e6103. doi: 10.1371/journal.pone.0006103 (PMC2699539; doi:10.1371/journal.pone.0006103)
Supplement: Table S1 — List of newspapers used in this study (0.06 MB DOC) [file pone.0006103.s001.doc]

**Table S1**. List of newspapers used in this study (n=47)

| Chicago Sun-Times |
| --- |
| Christian Science Monitor (Boston, MA) |
| Courier Mail (Queensland, Australia) |
| Daily News (New York) |
| Financial Times |
| Herald Sun (Melbourne, Australia) |
| Milwaukee Journal Sentinel (Wisconsin) |
| New Straits Times (Malaysia) |
| New York Times |
| Omaha World Herald (Nebraska) |
| San Francisco Chronicle |
| South China Morning Post (Hong Kong) |
| Scotland on Sunday |
| Seattle Times |
| St. Louis Post-Dispatch (Missouri) |
| St. Petersburg Times (Florida) |
| Sunday Herald Sun (Melbourne, Australia) |
| Sunday Telegraph (London) |
| Tampa Tribune (Florida) |
| The Advertiser |
| The Australian |
| The Boston Globe |
| The Boston Herald |
| The Daily Telegraph (London) |
| The Daily Telegraph (Sydney, Australia) |
| The Daily Yomiuri (Tokyo) |
| The Dominion (Wellington) |
| The Dominion Post (Wellington) |
| The Evening Post (Wellington) |
| The Guardian (London) |
| The Herald (Glasgow) |
| The Houston Chronicle |
| The Independent (London) |
| The Irish Times |
| The Jerusalem Post |
| The Nelson Mail (New Zealand) |
| The Observer |
| The Press (Christchurch) |
| The Scotsman |
| The Straits Times (Singapore) |
| The Sunday Telegraph (Sydney, Australia) |
| The Toronto Sun |
| The Weekend Australian |
| Times-Picayune (New Orleans, LA) |
| Toronto Star |
| USA Today |
| Washington Post |
