# Supplementary material for: Characteristics of Medical Research News Reported on Front Pages of Newspapers
Source: PLoS One. 2009 Jul 1;4(7):e6103. doi: 10.1371/journal.pone.0006103 (PMC2699539; doi:10.1371/journal.pone.0006103)
Supplement: Table S2 — List of journals associated with news based on mature research (0.11 MB DOC) [file pone.0006103.s002.doc]

**Table S2**. List of journals associated with news based on mature research (n=122)

| Academic Medicine |
| --- |
| Acta Paediatrica |
| Addiction |
| American Journal of Cardiology |
| American Journal of Clinical Nutrition |
| American Journal of Gastroenterology |
| American Journal of Hypertension |
| American Journal of Psychiatry |
| American Journal of Public Health |
| American Journal of Respiratory and Critical Care Medicine |
| American Journal of Roentgenology |
| American Psychologist |
| Annals of Internal Medicine |
| Annals of Oncology |
| Annals of Pharmacotherapy |
| Archives of Disease in Childhood |
| Archives of Environmental Contamination and Toxicology |
| Archives of Family Medicine |
| Archives of Internal Medicine |
| Archives of Pediatrics and Adolescent Medicine |
| Arteriosclerosis, Thrombosis and Vascular Biology |
| Australian and New Zealand Journal of Obstetrics and Gynaecology |
| Blood |
| BMJ |
| British Dental Journal |
| British Journal of Cancer |
| British Journal of Obstetrics and Gynaecology |
| British Journal of Psychology |
| Canadian Journal of Nursing Research |
| Cancer |
| Cancer Epidemiology, Biomarkers and Prevention |
| Cancer Research |
| Catheterization and Cardiovascular Interventions |
| Chemical Senses |
| Chinese Journal of Epidemiology (Zhonghua Liu Xing Bing Xue Za Zhi) |
| Circulation |
| Clinical and Diagnostic Laboratory Immunology |
| Clinical Cancer Research |
| Clinical Oncology |
| Canadian Medical Association Journal |
| Cornea |
| Developmental Psychology |
| Diabetes |
| Diabetes Care |
| EMBO Journal |
| Emerging Infectious Diseases |
| Environmental Health Perspectives |
| Epidemiology |
| European Heart Journal |
| FASEB |
| Fertility and Sterility |
| Genome Biology |
| Health Affairs |
| Health Services Management Research |
| Hormones and Behavior |
| Human Reproduction |
| Hypertension |
| Injury |
| International Journal for Quality in Health Care |
| International Journal of Cancer |
| International Journal of Cardiology |
| International Journal of STD & AIDS |
| Israel Medical Association Journal |
| JAMA |
| Journal of Agricultural and Food Chemistry |
| Journal of Clinical and Experimental Neuropsychology |
| Journal of Clinical Investigation |
| Journal of Clinical Oncology |
| Journal of Consulting and Clinical Psychology |
| Journal of Dentistry |
| Journal of Genetic Counseling |
| Journal of Health and Social Behavior |
| Journal of Health Economics |
| Journal of Human Virology |
| Journal of Immunoassay |
| Journal of Immunology |
| Journal of Neuroscience |
| Journal of Neuroscience Research |
| Journal of Pathology |
| Journal of Pediatrics |
| Journal of Personality and Social Psychology |
| Journal of Regenerative Medicine |
| Journal of Studies on Alcohol |
| Journal of Substance Abuse |
| Journal of the American Academy of Child and Adolescent Psychiatry |
| Journal of the American College of Cardiology |
| Journal of the American College of Nutrition |
| Journal of the American Dental Association |
| Journal of the American Dietetic Association |
| Journal of the National Cancer Institute |
| Journal of the Royal College of Physicians of London |
| Journal of Virology |
| Lancet |
| Med J Aust |
| Nature |
| Nature Biotechnology |
| Nature Genetics |
| Nature Medicine |
| N Engl J Med |
| Neurology |
| New Zealand Medical Journal |
| Occupational and Environmental Medicine |
| Pediatric Annals |
| Pediatrics |
| Perspectives on Sexual and Reproductive Health |
| Pharmacogenetics |
| Physics in Medicine and Biology |
| Physiology and Behavior |
| PNAS |
| Postgraduate Medical Journal |
| Preventive Medicine |
| Psychosomatic Medicine |
| Public Health Reports |
| Quarterly Journal of Experimental Psychology. A, human experimental psychology |
| Radiology |
| Rheumatic Diseases Clinics in North America |
| Science |
| Sexually Transmitted Diseases |
| Spine |
| Thoracic and Cardiovascular Surgeon |
| Thorax |
| Vaccine |
