# Supplementary material for: Characteristics of Medical Research News Reported on Front Pages of Newspapers
Source: PLoS One. 2009 Jul 1;4(7):e6103. doi: 10.1371/journal.pone.0006103 (PMC2699539; doi:10.1371/journal.pone.0006103)
Supplement: Table S3 — List of journals associated with news based on preliminary findings (0.10 MB DOC) [file pone.0006103.s003.doc]

**Table S3**. List of journals associated with news based on preliminary findings (n=113)

| Acta Oncologica |
| --- |
| Addiction |
| AIDS |
| Alcohol |
| American Journal of Clinical Nutrition |
| American Journal of Health Promotion |
| American Journal of Industrial Medicine |
| American Journal of Infection Control |
| American Journal of Medical Genetics |
| American Journal of Neuroradiology |
| American Journal of Psychiatry |
| American Journal of Public Health |
| Annals of Allergy, Asthma & Immunology |
| Annals of Epidemiology |
| Annals of Internal Medicine |
| Annals of Thoracic Surgery |
| Archives of Disease in Childhood |
| Archives of Internal Medicine |
| Archives of Neurology |
| Archives of Ophthalmology |
| Archives of Sexual Behaviour |
| AWHONN Lifelines (Association of Women’s Health, Obstetric and Neonatal Nursing) |
| Biological Psychiatry |
| Blood |
| BMJ |
| British Dental Journal |
| Cancer |
| Cancer Causes and Control |
| Cancer Epidemiology, Biomarkers and Prevention |
| Cancer Research |
| Carcinogenesis |
| Catheterization and Cardiovascular Intervention |
| Circulation |
| Clinical and Experimental Allergy |
| Clinical and Experimental Pharmacology and Physiology |
| Clinical Cancer Research |
| Clinical Infectious Diseases |
| Clinical Neuropsychologist |
| Clinical Oncology (Royal College of Radiologists) |
| Clinical Pharmacology & Therapeutics |
| Contraception |
| Development |
| Diabetes Care |
| Emerging Infectious Diseases |
| Endocrinology |
| Environmental Health Perspectives |
| Environmental Science & Technology |
| European Heart Journal |
| European Journal of Cancer |
| European Journal of Clinical Nutrition |
| Gynaecologic Oncology |
| Health Affairs |
| Health Service Journal |
| Hepatology Research |
| Human Reproduction |
| International Journal of Cardiology |
| International Journal of Impotence Research |
| International Journal of Radiation Oncology, Biology, Physics |
| International Journal of STD & AIDS |
| International Psychogeriatrics |
| JAMA |
| Journal of Acquired Immune Deficiency Syndromes |
| Journal of Applied Animal Welfare Science |
| Journal of Bone and Joint Surgery. British Volume |
| Journal of Cardiac Failure |
| Journal of Clinical Oncology |
| Journal of Clinical Pharmacology |
| Journal of Clinical Psychiatry |
| Journal of Geriatric Psychiatry and Neurology |
| Journal of Heart and Lung Transplantation |
| Journal of Human Nutrition and Dietetics |
| Journal of Marital and Family Therapy |
| Journal of Molecular Neuroscience |
| Journal of Neurochemistry |
| Journal of Neuroscience |
| Journal of Neuroscience Research |
| Journal of Physiology |
| Journal of Sex & Marital Therapy |
| Journal of Telemedicine and Telecare |
| Journal of the Air and Waste Management Association |
| Journal of the American Academy of Child and Adolescent Psychiatry |
| Journal of the National Cancer Institute |
| Journal of Thoracic and Cardiovascular Surgery |
| Journal of Virology |
| Journal of Women’s Health & Gender-based Medicine |
| Lancet |
| Managed Care Quarterly |
| Medicine and Science in Sports and Exercise |
| Menopause |
| Nature |
| N Engl J Med |
| Neuropsychologia |
| Neuropsychopharmacology |
| Nutritional Neuroscience |
| New Zealand Medical Journal |
| Obesity Research |
| Obstetrics and Gynecology |
| Pediatric Infectious Disease Journal |
| Pediatric Physical Therapy |
| Pediatric Research |
| Pediatrics |
| Pharmacology and Therapeutics |
| Pharmacology, Biochemistry, and Behavior |
| Physiology and Behavior |
| PNAS |
| Prostate |
| Psychosomatic Medicine |
| Reproductive Biomedicine Online |
| Reproductive Toxicology |
| Science |
| Stem Cells |
| Tobacco Control |
| Transplantation |
